# Network theoretic analysis of JAK/STAT pathway and extrapolation to drugs and viruses including COVID-19

**DOI:** 10.1038/s41598-021-82139-x

**Published:** 2021-01-28

**Authors:** Arindam Banerjee, Rudra Prosad Goswami, Moumita Chatterjee

**Affiliations:** 1 Department of Mathematics, Ramakrishna Mission Vivekananda Educational and Research Institute, Belur, India; 2 Department of Rheumatology, All India Institute of Medical Sciences, New Delhi, India; 3 Department of Mathematics and Statistics, Aliah University, Kolkata, India

**Keywords:** Cellular signalling networks, Data mining, Databases, Rheumatology, Rheumatoid arthritis, Viral infection

## Abstract

Whenever some phenomenon can be represented as a graph or a network it seems pertinent to explore how much the mathematical properties of that network impact the phenomenon. In this study we explore the same philosophy in the context of immunology. Our objective was to assess the correlation of “size” (number of edges and minimum vertex cover) of the JAK/STAT network with treatment effect in rheumatoid arthritis (RA), phenotype of viral infection and effect of immunosuppressive agents on a system infected with the coronavirus. We extracted the JAK/STAT pathway from Kyoto Encyclopedia of Genes and Genomes (KEGG, hsa04630). The effects of the following drugs, and their combinations, commonly used in RA were tested: methotrexate, prednisolone, rituximab, tocilizumab, tofacitinib and baricitinib. Following viral systems were also tested for their ability to evade the JAK/STAT pathway: Measles, Influenza A, West Nile virus, Japanese B virus, Yellow Fever virus, respiratory syncytial virus, Kaposi’s sarcoma virus, Hepatitis B and C virus, cytomegalovirus, Hendra and Nipah virus and Coronavirus. Good correlation of edges and minimum vertex cover with clinical efficacy were observed (for edge, rho =  − 0.815, R^2^ = 0.676, p = 0.007, for vertex cover rho =  − 0.793, R^2^ = 0.635, p = 0.011). In the viral systems both edges and vertex cover were associated with acuteness of viral infections. In the JAK/STAT system already infected with coronavirus, maximum reduction in size was achieved with baricitinib. To conclude, algebraic and combinatorial invariant of a network may explain its biological behaviour. At least theoretically, baricitinib may be an attractive target for treatment of coronavirus infection.

## Introduction

Whenever some phenomenon can be represented as a graph or a network it seems pertinent to explore how much the mathematical properties of that network impact the phenomenon. This has been done in various branches of physical biological and social sciences. To give an example, one such very important development is the topic of chemical graph theory^1^. In this article we explore the same philosophy in the context of medicine. We study how the effectiveness of various medications and viruses are related to the "size" of the human Janus Kinase/Signal Transducers and Activators of Transcription (JAK/STAT) pathway network. Now there are various measures of size for graphs or networks. Here we experiment with two such, namely the number of edges and minimum size of a vertex cover. While number of size is a crude combinatorial measure the other one is a subtler invariant with some algebraic interpretation. For any graph the minimum size of a vertex cover is the height of the underlying edge ideal which is basically the dimension of the related algebraic variety^2^. It turns out that for our case the number of edges is a good quantitative measure of effectiveness and minimum size of a vertex cover is a good qualitative measure.

Biochemical networks, such as signalling pathways, like the JAK/STAT pathway, are protein–protein interaction networks. These interaction networks represent dynamical system, which under perturbation, may either transition into a different stability point or collapse entirely^3^.

Standard treatment of immunological diseases like rheumatoid arthritis (RA) is dependent on immunomodulatory drugs, like, methotrexate or tocilizumab, etc^4^. However, guidance of use of these drugs is from expert opinion and randomised controlled trials. Majority of these drugs work on certain subcellular signalling networks, like the JAK/STAT pathway, which is one of the most important pathways, for the inflammatory process. Blocking this pathway results in reduction of inflammatory cascade. Many drugs work on many different levels on this pathway and there is no clear-cut theoretical understanding to guide, which drugs should work best on this pathway and result in disease remission. The mechanism of immunomodulatory drugs is to inhibit these pathways, and therefore it comes to reason that after introduction of these drugs in the system, the size of the pathway or signalling network should be reduced and this should parallel the clinical outcome. On the other hand various viral infections evade the host human anti-viral defence mechanisms through certain proteins, which inhibit inflammatory cascades especially the JAK/STAT pathways. Therefore similarly as in case of the effect of drugs, ability of these viruses to reduce the network size could be paralleled by their ability to successfully evade the host defence mechanisms for prolonged periods of time and result in chronicity. Certain viruses like Corona virus cause end organ damage by cytokine storm, often employing the host JAK/STAT pathway^5^.

Our objectives were to assess the correlation of “size” of the JAK/STAT network with treatment effect in rheumatoid arthritis (RA), phenotype of viral infections and effect of immunosuppressive agents on a system infected with the coronavirus.

## Results

In the fully annotated human JAK/STAT pathway from KEGG, in an uninterrupted system, initially there are 868 edges and minimum size of a vertex cover is 83. Table 1 lists the full set of drugs used in RA both alone and in combination with methotrexate. It also lists clinical efficacy of the drugs. In the second panel of the table effects of different viral evasion mechanisms on this pathway in terms of size of the way is depicted.

**Table 1:** Effect of different drugs and viral evasion mechanisms on the network size of the human Janus Kinase/Signal Transducers and Activators of Transcription (JAK/STAT) pathway.

Drugs used in rheumatoid arthritis								Viral systems							
Alone	Edge	Vertex cover	ACR 20	+ Methotrexate	Edge	Vertex cover	ACR 20	Virus	Edge	Vertex cover	Type	Virus	Edge	Vertex cover	Type
Methotrexate	569	18	39.70	–	–	–	–	Measles	574	18	Acute	KSV	460	16	Chronic
Tocilizumab	589	18	59	Tocilizumab	559	18	55.80	Dengue	569	18	Acute	HBV	460	16	Chronic
Ritux	584	18	65	Ritux	544	17	54	Influenza A	574	18	Acute	CMV	584	18	Acute
Pred	527	17	51	Pred	499	17	70.63	EBV	554	18	Acute	Hendra/Nipah	569	18	Acute
Tofacitinib	473	16	65.70	Tofacitinib	447	16	75.40	RSV	508	17	Acute	Corona	559	18	Acute
Baricitinib	473	16	77	Baricitinib	447	16	78	WNV/JBV/YFV	584	18	Acute	HCV	460	16	Chronic

ACR 20 responses are expressed as percentages.

ACR 20: American College of Rheumatology 20 response; CBV: cytomegalovirus; EBV: Epstein Barr virus; HBV: hepatitis B virus; HCV: hepatitis C virus; JBV: Japanese B virus; KSV: Kaposi’s sarcoma virus; RSV: respiratory syncitial virus; WNV: West Nile virus; YFV: yellow fever virus.

**Figure 1:**
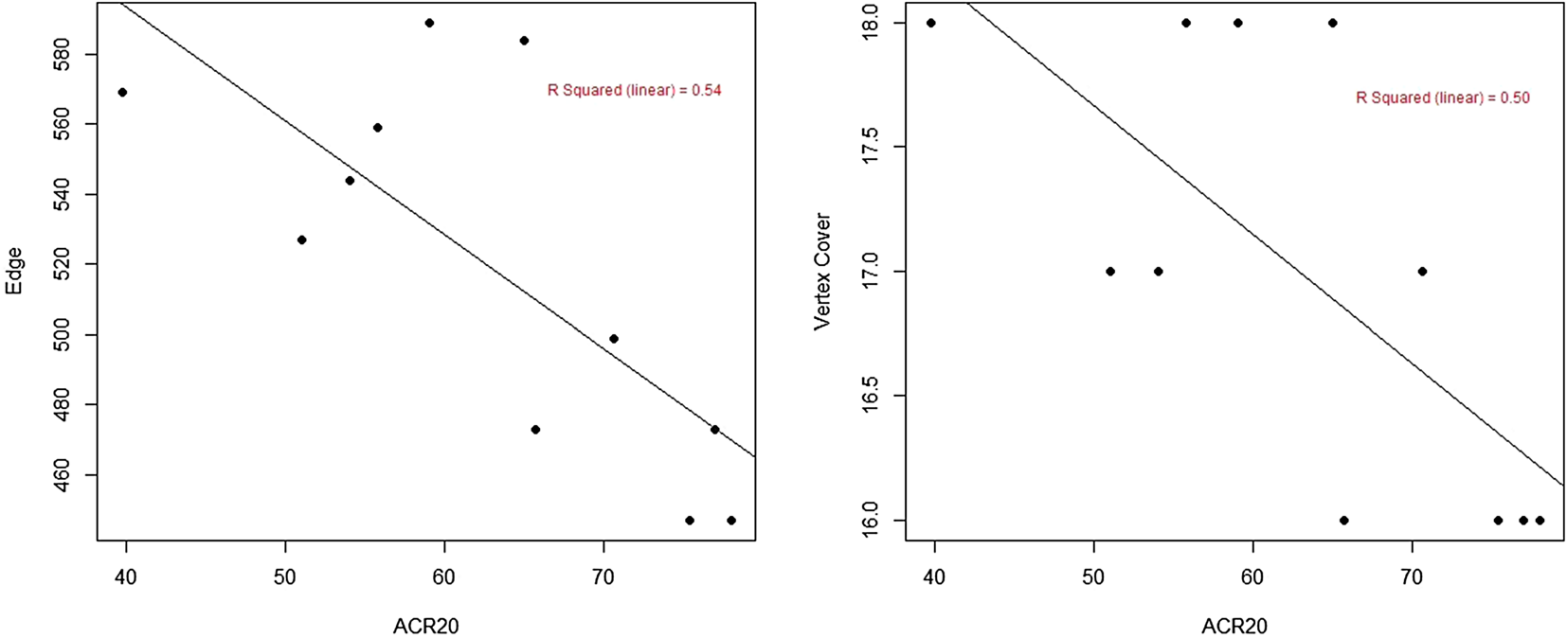
Represents scatter plot of treatment response (American College of Rheumatology 20 response (ACR 20), x-axis) with edges and minimum vertex cover (y-axes). The data points represent different drugs. The results are obtained with deletion of rituximab from the data set. The linear regression coefficient for edges is 0.676 and that for the minimum vertex cover is 0.635.

Spearman’s correlations between ACR20 with both edge and minimum vertex cover were significant (for both edge and vertex cover, rho = − 0.708, p = 0.015). Since rituximab is not a classic JAK/STAT pathway inhibitor, the results were re-run excluding rituximab. Correlations with ACR20 after excluding rituximab were improved (for edge, rho = − 0.815, p = 0.007, for vertex cover rho = − 0.793, p = 0.011). In the scatter plot also, after excluding rituximab linear regression coefficient (R^2^) was 0.676 for edge and 0.635 for minimum vertex cover (Fig. 1).

In the viral systems both edges and vertex cover were associated with acuteness of viral infections: acute (n = 12) vs. chronic (n = 3) respectively, edges 567.7 ± 21.2 vs. 460 ± 0, p = 0.002; vertex cover: 17.91 ± 0.28 vs 16 ± 0, p = 0.002.

**Figure 2:**
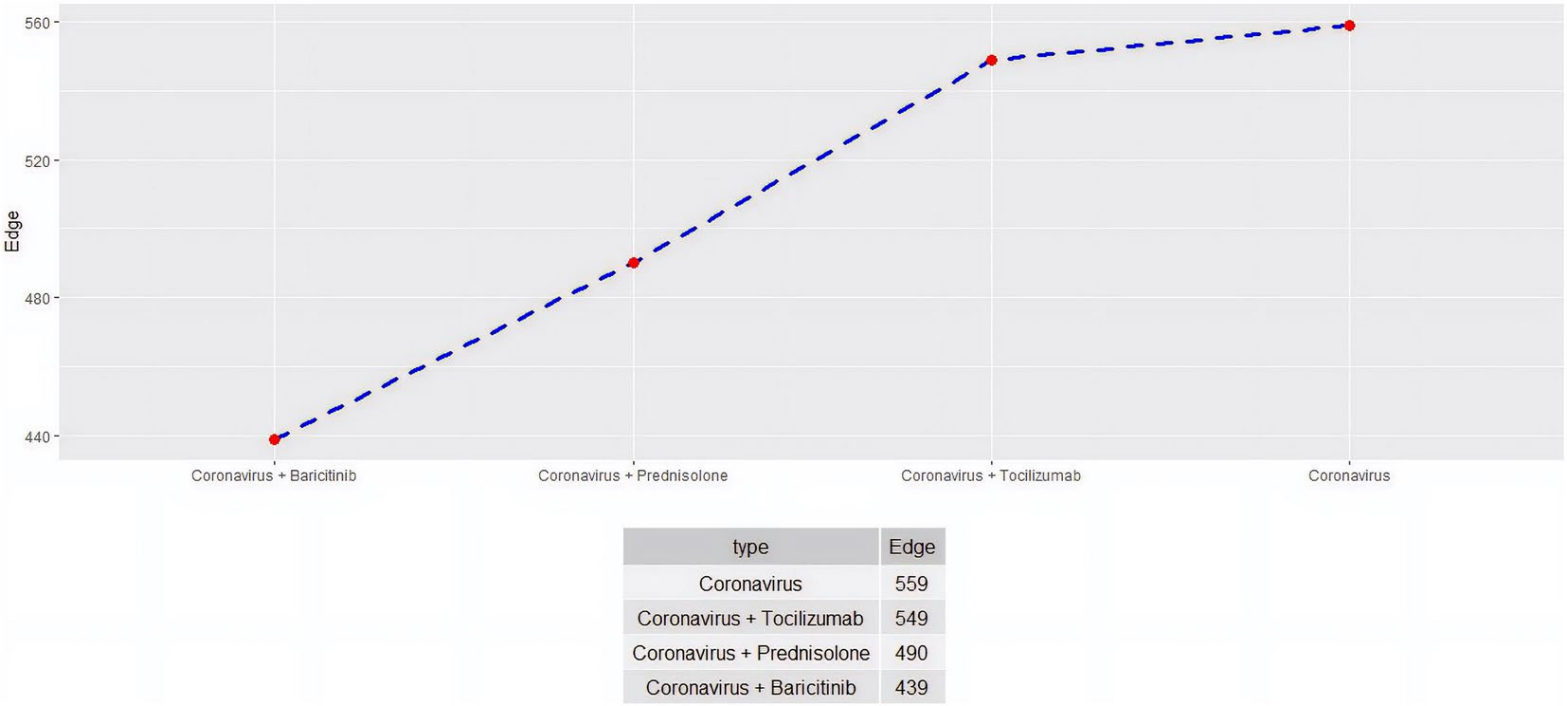
Network size of the human Janus Kinase/Signal Transducers and Activators of Transcription (JAK/STAT) pathway network infected with coronavirus. The system is further tested in terms of size after introduction of immunosuppressive drugs, namely, prednisolone, tocilizumab and baricitinib. In this figure the number of edges is depicted. Maximum reduction of network edges is obtained with baricitinib, intermediate with glucocorticoids and least with tocilizumab.

**Figure 3:**
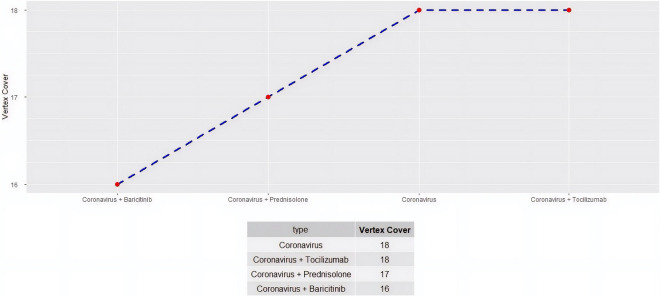
Network size of the human Janus Kinase/Signal Transducers and Activators of Transcription (JAK/STAT) pathway network infected with coronavirus. The system is further tested in terms of size after introduction of immunosuppressive drugs, namely, prednisolone, tocilizumab and baricitinib. In this figure the minimum size of vertex cover is depicted. Similar to Fig. 2, maximum reduction of network size is obtained with baricitinib again.

In the JAK/STAT system already infected with coronavirus the effect of adding the following drugs were tested: prednisolone, baricitinib and tocilizumab (Fig. 2 for edge and Fig. 3 for minimum vertex cover). Almost no reduction in size of the network was seen with tocilizumab, whereas maximum reduction was achieved with baricitinib.

## Discussion

This work indicates that further exploration with other networks might help to understand relative effectiveness of many other medications and viruses. In case these measures work similarly as in here in some other cases also then one can consider these as important invariants for further use. This also gives motivation to explore how various other invariants related to networks or graphs like chromatic numbers, various Betti numbers, entropy etc. summarises the biological phenomenon. There are various computer softwares for computing such invariants and their introduction is expected to develop various new dimensions in the research of medical informatics.

In purely mathematical terms already building a dictionary between algebra and combinatorics is being under progress in the study of graph vis-à-vis their edge ideals^2^. This work underscores that algebraic invariants of biological networks may control the qualitative property of the underlying phenomenon while the combinatorial invariant controls the quantitative.

In the first two experiments we observed that these size invariants correlate quantitatively well with biological phenomena, i.e., treatment effect of drugs in rheumatoid arthritis and acuteness of viral illnesses with competency of viral evasion mechanisms. These results seem to be highly specific to the JAK/STAT pathway. Rituximab is not a classical JAK/STAT pathway inhibitor and only effects on this drug on this pathway are from cancer studies. However, after restricting the study to only pure JAK/STAT pathway inhibitors, the correlations were near perfect with good linear regression coefficient.

In the final experiment, after introduction of the immunosuppressive drugs in a JAK/STAT system already infected with the coronavirus, we observed that significant reduction of size was achieved with baricitinib only. Coronavirus related serious pulmonary injury is postulated cytokine storm closely related to JAK/STAT system. Several drugs are being tried, most notably tocilizumab^6^. However, our experiments demonstrate that, at least theoretically, immunosuppressive effect of the tyrosine kinase inhibitor baricitinib supersedes that of prednisolone and tocilizumab and hence baricitinib may be more effective in the current scenario. During the process of peer-review results from two clinical trials were published, which, similar to our hypothesized results, showed that baricitinib-treated patients with coronavirus related cytokine storm had a marked reduction in serum levels of several cytokines and rapid recovery in circulating T and B cell frequencies, and another trial reported that In patients with moderate to severe coronaviral pneumonia combination of baricitinib with corticosteroids was associated with greater pulmonary functional improvement compared to corticosteroids alone^7,8^.

Limitations of our study are that, it works with only one network and a limited number of noises. But, these results warrant further study. Further treatment models on diverse networks need to be tested.

Finally, our proposed technique will allow us to theoretically predict suitable targets with putative efficacy in various clinical scenarios, allowing targeted and biologically tailored clinical trial validation.

## Methods

The human JAK/STAT pathway is well detailed and fully annotated protein–protein interaction network is available from the Kyoto Encyclopedia of Genes and Genomes (KEGG) (http://www.genome.jp/kegg/)^9,10^. KEGG pathway is a collection that includes well-ordered pathways mined from textbooks, literature, other databases and expert knowledge^11–13^. We extracted the JAK/STAT pathway from Kyoto Encyclopedia of Genes and Genomes (KEGG, hsa04630). The pathway was downloaded as KGML files. In the next a mathematical graph representation of the pathway was done. In the graph representation, a numbered vertex represents each protein and interactions between the proteins are represented by edges. The adjacency list was generated with the software R^14^. The KEGG pathways have directional components as well. We view a network as a directed graph and note the number of edges and the minimum size of a vertex cover of the underlying undirected graph (a vertex cover in a graph is a set of vertices such that given any edge at least one of its vertex belongs to that set; for example for the graph G with V(G) = {a,b,c,d} and E(G) = {ab,ac,ad} both {a} and {b, c, d} are vertex covers but {b, c} is not a vertex cover). For each network if one deletes a set of vertices then all the edges that have at least one vertex in that set also deleted and we are left with a smaller network. The considered network has 155 nodes, 868 edges and minimum size of a vertex cover is 83. We know that certain vertices (serially numbered from 59 to 99) are network "entry" points of this network (through which signals enter the network from environment). Each of the medications and viruses we experiment with acts by deleting some nodes of this network. Now as directed edges in this network imply "flow of signals", if after deleting some nodes it turns our that there are some nodes where no signal is entering, we delete that node as long as it is not an entry point; as this implies that it will not be part of our biological process anymore. Now deletion of such nodes may further create some nodes where no signals enter and we delete them too (as long as they are not entry point nodes). We continue this way until no more such nodes are left. And for each case we observe the number of edges and minimum size of a vertex cover. For doing this we use R programming software^14^. We first calculated the size of the original unperturbed network. Then the effects of the following drugs (respective targets), and their combinations, commonly used in RA were tested: methotrexate (STAT 1 (hsa6772) and STAT 5 (hsa6776)), prednisolone (interleukin 2 receptor subunit alpha (has 3559), JAK3 (hsa3718)), rituximab (STAT3 (hsa6774)), tocilizumab (interleukin-6 receptor (hsa3570)), tofacitinib (JAK1 (hsa3716), hsa3718) and baricitinib (hsa3716, JAK2 (hsa3717))^15–17^. Following viral systems were also tested for their ability to evade the immune system through interference with the JAK/STAT pathway: Measles: interferon alpha receptor 1 (hsa3454), hsa6772; Influenza A: hsa3454, hsa6774; hsa3454, hsa6772, interferon gamma receptors 1 and 2 (hsa3459, hsa3460); West Nile virus (WNV), Japanese B virus (JBV) and Yellow Fever virus (YFV): hsa6772; dengue virus: hsa6772, STAT2 (has 6773); respiratory syncytial virus (RSV): Tyk2 (hsa7297), CREB binding protein (hsa1387), hsa6772, hsa6773; Kaposi’s sarcoma virus (KSV): hsa3716, hsa7297, hsa6773; Hepatitis B and C virus (HBV and HCV): hsa3716, hsa7297, hsa6772); cytomegalovirus (CMV): hsa6772, interferon regulatory factor 9 (hsa10379); Hendra and Nipah virus (HV and NV): hsa6772, hsa6773; Coronavirus: hsa3454, hsa6772, hsa6773^18^.

In addition to calculation of network sizes in these perturbed networks, we also accessed the treatment responses of the above-mentioned drugs in RA in terms of the American College of Rheumatology 20 (ACR 20) response^19–28^.

The treatment responses (ACR20 numerical values) were correlated with the size invariants of the networks. The median sizes of the networks were compared between the viruses, which cause acute infections (acute: measles, influenza A, WNV, JBV, YFV, dengue virus, CMV, HV, NV, RSV, EBV, coronavirus; chronic: KSV, HBV, HCV).
